# Temporal Dissociation Between Intravascular Albumin Mass and Transcapillary Escape Dynamics in Sepsis: A Longitudinal Characterization of Albumin Homeostasis Using Routine Laboratory Parameters

**DOI:** 10.3390/jcm15062427

**Published:** 2026-03-22

**Authors:** Gianni Turcato, Arian Zaboli, Lucia Filippi, Michael Maggi, Alessandra Eugenia Bionda, Fabrizio Lucente, Alberto Caregnato, Daniela Milazzo, Paolo Ferretto, Lorenzo Ghiadoni, Christian J. Wiedermann

**Affiliations:** 1 Department of Internal Medicine, Intermediate Care Unit, Hospital Alto Vicentino (AULSS7), 36014 Santorso, Italy; 2 Department of Health Sciences, UniCamillus-Saint Camillus International University of Health Sciences, 00131 Rome, Italy; 3 Health Professions Management, South Tyrolean Health Authority (SABES-ASDAA), 39100 Bolzano, Italy; 4 Department of Clinical and Experimental Medicine, University of Pisa, 56126 Pisa, Italy; 5 Institute of General Practice and Public Health, Claudiana College of Health Professions, 39100 Bolzano, Italy

**Keywords:** net intravascular albumin balance, sepsis-related vasoplegia, sepsis-related capillary leak

## Abstract

**Background:** In sepsis, albumin homeostasis is altered by capillary leak and recovery mechanisms (synthesis and interstitial–lymphatic return), which are difficult to capture clinically. A joint evaluation of ratio-based escape dynamics and mass-based balance may clarify their temporal interplay in sepsis. **Methods**: In a prospective longitudinal cohort of 389 sepsis patients admitted to an intermediate medical care unit, serial daily sampling (up to five reassessments; 1897 observations) was used to derive: a transcapillary escape rate (TER)-like index from the hourly percent change in the albumin/hemoglobin ratio and net albumin leakage (NAL) from changes in intravascular albumin mass normalized to plasma volume. Indices were analyzed as continuous measures and by sign-defined combined states. Generalized estimating equations (GEE), patient-level slope analyses, and state-transition analyses were performed. Baseline SOFA and 30-day mortality were explored as effect modifiers. **Results:** The TER-like index peaked early (median +0.34%/h at day 1) and declined to negative values by day 5 (median −0.07%/h), with the TER-like index > 0 decreasing from 65.3% to 45.8%. NAL was frequently negative and heterogeneous (NAL ≥ 0 in 37.5% of observations). TER-like index and NAL were inversely correlated (ρ = −0.54; *p* < 0.001). In GEE, the TER-like index was associated with NAL (β = −7.46 g/L per 1%/h; 95% CI −8.69 to −6.22; *p* < 0.001) with time-varying effects (interaction *p* < 0.01). The dissociation state (TER-like index ≤ 0 with NAL < 0) increased from 2.3% at day 1 to 22.6% at day 5 (*p* < 0.001). **Conclusions:** TER-like index escape dynamics attenuate early; however, net intravascular albumin balance often remains negative, supporting temporal uncoupling between “leak” and recovery. An integrated TER-like/NAL assessment offers a pragmatic framework for the clinical phenotyping of albumin homeostasis using routine laboratory data.

## 1. Introduction

Sepsis is associated with high mortality, and in-hospital mortality in septic shock exceeds 30–40% despite decades of research [1,2,3,4]. This persistent inability to improve patient outcomes likely reflects an incomplete understanding of the underlying pathophysiology, resulting in a lack of informative biomarkers and therapeutic strategies often driven by simplified assumptions rather than a direct appraisal of the biological processes involved [5,6,7]. A paradigmatic example is fluid resuscitation, a cornerstone of sepsis management, based on the assumption that vasoplegia and hypotension require intravascular volume expansion to preserve organ perfusion [8,9]. However, this strategy is applied within a complex pathophysiological context in which fluid and plasma protein distribution is profoundly altered and cannot be reduced to a simple volume deficit [10,11].

In this context, two physiologically well-established yet clinically underappreciated mechanisms emerge as central to sepsis pathophysiology: capillary permeability, altered by inflammatory and endothelial injury processes, which promotes the extravasation of protein-rich fluid into the interstitium [11,12,13], and the mechanisms responsible for restoring plasma proteins to the intravascular compartment through lymphatic drainage and interstitial recovery pathways [14,15]. Both processes critically involve serum albumin, the most abundant plasma protein and a key determinant of vascular–interstitial homeostasis, which is profoundly dysregulated in sepsis [6,10,16,17]. Inflammatory activation not only enhances intravascular protein loss but also impairs restorative pathways, shifting the equilibrium toward net albumin depletion and sustained interstitial edema. Nevertheless, the longitudinal dynamics of albumin homeostasis remain poorly characterized in clinical populations, particularly in patients with sepsis in intermediate-care units with moderate disease severity.

The gold-standard assessment of albumin kinetics, based on radioactive tracer studies measuring transcapillary escape rate (TER) and synthesis rate, is resource-intensive and unsuitable for routine clinical application [18]. Consequently, albumin evaluation typically relies on static concentration thresholds, overlooking the dynamic balance among leakage, synthesis, and recovery. Serial routine laboratory parameters may allow indirect evaluation of albumin dynamics. The albumin-to-hemoglobin ratio (ALB/HB), with hemoglobin as an intravascular reference, provides a volume-adjusted marker of relative protein shifts and selective albumin loss [18,19,20]. Intravascular albumin mass (IVAM), derived from albumin concentration and estimated plasma volume, quantifies the circulating pool, whereas net albumin leakage (NAL) integrates the balance among extravasation, synthesis, lymphatic return, and other losses [18,19,20]. Integrating ratio- and mass-based albumin indices may provide a pragmatic window into the dynamic equilibrium between capillary loss and protein restoration, enabling mechanistic phenotyping and mechanism-oriented therapeutic strategies.

Classical TER quantifies the unidirectional fractional rate of albumin transfer from the intravascular to the interstitial compartment (%/h), with normal values of approximately 5–6%/h in healthy individuals [21,22]. The TER-like index derived in the present study approximates this construct using the hourly percentage change in the ALB/HB ratio, but—unlike classical TER—reflects net changes in the relative intravascular albumin pool rather than unidirectional flux and cannot isolate permeability from synthesis, recovery, or volume redistribution effects.

This study aimed to longitudinally characterize albumin homeostasis in patients with sepsis in the IMCU using routine laboratory-derived indices. We sought to disentangle transcapillary albumin escape from net intravascular balance by jointly evaluating a transcapillary escape ratio-based kinetic index (TER-like index) and NAL, capturing their dynamic interplay and potential uncoupling. We further examined combined TER-like index-NAL trajectories to define distinct albumin homeostasis phenotypes and assess their associations with baseline severity, 30-day mortality, and temporal transitions, with the goal of establishing a pragmatic framework for routine albumin kinetics assessment in sepsis.

## 2. Materials and Methods

### 2.1. Study Design and Patients

This prospective longitudinal observational study was conducted in the medical IMCU of the Alto Vicentino Hospital, Santorso (Italy), between 1 September 2023, and 31 March 2025. We included all adult patients admitted from the emergency department (ED) with sepsis, as defined by the Sepsis-3 criteria, as suspected or documented infection associated with an increase in the Sequential Organ Failure Assessment (SOFA) score ≥2 points [23]. The exclusion criteria were age <18 years, pregnancy, transfer from wards other than the ED, postoperative or post-traumatic sepsis within the preceding month, ED length of stay > 6 h, receipt of >1000 mL crystalloids in the 3 h before admission, administration of exogenous albumin or synthetic colloids during the observation period, terminal illness with life expectancy < 3 months, and immediate need for intensive organ support, either initiated in the ED or withheld due to treatment limitations. Patients receiving albumin or colloids were excluded to avoid confounding of endogenous albumin kinetics, in line with current recommendations discouraging routine colloid use in sepsis [24,25].

The study was approved by the local ethics committee (protocol number: 406, 3 October 2023) and conducted in accordance with the principles of the Declaration of Helsinki. Written informed consent was obtained from all participants or their legal representatives prior to enrollment.

### 2.2. Study Protocol and Data Collection

#### 2.2.1. Baseline Assessment (t_0_)

At enrollment, demographic and clinical data were collected, including age, sex, anthropometrics (weight, height, body mass index), and comorbidities used to calculate the Charlson Comorbidity Index (CCI). Admission laboratory tests included complete blood count with differential, serum electrolytes, renal and hepatic function markers, coagulation profile (prothrombin-activated time-international normalized ratio, activated partial thromboplastin time, fibrinogen, and D-dimer), inflammatory markers (C-reactive protein and procalcitonin), and serum albumin. Arterial blood gas analysis with lactate was obtained via an arterial catheter to enable standardized serial sampling; a urinary catheter was placed for accurate urine output and fluid balance monitoring. Based on the admission data, SOFA and Acute Physiology and Chronic Health Evaluation II (APACHE II) scores were calculated.

#### 2.2.2. Serial Reassessments (t_1_–t_5_)

From day 1 to hospital day 5, baseline laboratory parameters were repeated daily at 08:00 (±30 min) to minimize circadian variability. Samples were collected via an arterial catheter. At each time point, laboratory results, arterial blood gas with lactate, and 24 h cumulative fluid balance were recorded.

#### 2.2.3. Outcome Assessment

The primary outcome was 30-day all-cause mortality, which was determined from hospital records or post-discharge telephone follow-up.

### 2.3. Derivation of Albumin Kinetic Parameters

The dynamic balance between transcapillary albumin loss and intravascular restoration was characterized using two complementary metrics, NAL and a TER-like index, both derived from serial laboratory measurements.

NAL was defined as the net change in intravascular albumin mass per unit of time between two consecutive reassessments, expressed in g/L per day:

$${NAL}_{t0\to tx}\left(g/L/day\right)=\frac{\left(\frac{{IVAM}_{tx}-{IVAM}_{t0}}{PV}\right)}{∆t}$$

where PV represents plasma volume between time points, and Δ*t* is the time interval. Plasma volume at each time point (PV_t_) was calculated as follows:

$${PV}_{t}=BV\times \left(1-HCT\right)\;\times \;0.91$$

where BV is the total blood volume estimated at baseline using Nadler’s sex-specific anthropometric equations, HCT_t_ is the hematocrit (fraction), and 0.91 adjusts for whole-body hematocrit distribution. This approach is widely applied in physiological mass balance and albumin kinetic studies.

The intravascular albumin mass at time t was defined as follows:

$${IVAM}_{t}\left(g\right)={ALB}_{t}\left(g/L\right)\;\times \;PV_{t}(L)$$

where ALB_t_ is the plasma albumin concentration. Unlike the concentration alone, IVAM reflects the absolute circulating albumin pool and reduces the confounding effects of dilution.

Therefore, NAL reflects the net balance between transcapillary loss, intravascular restoration, and albumin synthesis over time.

Classically, the TER quantifies the unidirectional fractional flux of albumin from the intravascular to the interstitial compartment (%/h) using radioactive tracer techniques with compartmental modeling [21,22]. As these methods are impractical in routine care, we derived a ratio-based kinetic index from serial laboratory data. To minimize dilutional effects, albumin was normalized to hemoglobin, an intravascular reference protein. For each time point:

$$R_{t}=\frac{A{LB}_{t}\left(g/dL\right)}{{HB}_{t}(g/dL)}$$

where ALB_t_ and HB_t_ represent serum albumin and hemoglobin concentrations, respectively. The TER-like index was defined as the hourly percentage change in this ratio between consecutive reassessments:

$${TER}_{t0\to tx}\left(\%/h\right)={((R}_{tx}-R_{t0})/R_{t0})\;\times \;100/∆t$$

Positive values indicate increased relative transcapillary albumin loss; values near zero indicate stability; and negative values reflect relative albumin recovery, reduced extravasation, or potential methodological influences. A ratio-based TER-like index was adopted, acknowledging that it does not replicate tracer-derived TER measurements. Although radioactive techniques remain the reference standard for quantifying unidirectional albumin flux, they are not applicable in routine clinical practice. Although this index cannot disentangle capillary permeability from synthesis and return mechanisms, it reflects the relative amount of albumin no longer retained within the vascular compartment compared with hemoglobin, which remains intravascular. In this sense, it represents a coarse yet clinically meaningful estimate of net protein loss, of which albumin is the principal component, derived from longitudinal sepsis real-world data. The TER-like index and classical TER are expected to be directionally concordant, but their numerical scales and physiological referents differ fundamentally; this distinction is important when interpreting TER-like index values against the established TER reference range of approximately 5–6%/h in healthy individuals [24,25].

### 2.4. Index Interpretation and State Classification

For an integrated analysis of albumin homeostasis, the TER-like index and NAL were evaluated as continuous variables and categorized by sign. Each observation was classified into four states according to the TER-like index (>0 vs. ≤0) and NAL (≥0 vs. <0):

- State 1 (TER-like index > 0, NAL < 0): Relative albumin decline with net intravascular depletion, consistent with ongoing differential loss. This pattern is most prevalent in the early phase of sepsis, when endothelial injury and glycocalyx disruption drive active transcapillary albumin extravasation. Hepatic albumin synthesis is concurrently suppressed by the acute-phase response, while lymphatic recovery has not yet compensated for the rate of loss. This state represents the canonical early sepsis albumin phenotype.
- State 2 (TER-like index ≤ 0, NAL < 0): Stable or improving ratio despite persistent mass depletion, suggesting dissociation between relative distribution and absolute pool reduction. This dissociation state reflects a clinical phase in which the ALB/HB ratio has stabilized—suggesting that relative albumin escape has attenuated—yet the absolute intravascular albumin pool continues to decline. Possible mechanisms include persistent impairment of hepatic synthesis, insufficient lymphatic recovery, or proportional losses of both albumin and hemoglobin, maintaining ratio stability despite ongoing mass depletion.
- State 3 (TER-like index ≤ 0, NAL ≥ 0): ‘Ratio stabilization with net albumin recovery, compatible with restoration of intravascular balance. This pattern suggests concurrent attenuation of transcapillary escape and adequate compensatory mechanisms—resumed synthesis, enhanced lymphatic return, or interstitial washdown—sufficient to achieve positive net albumin balance. It represents the most favorable homeostatic state and is expected to predominate during clinical recovery.
- State 4 (TER-like index > 0, NAL ≥ 0): ‘Relative ratio decline with mass increase, a physiologically uncommon pattern. This combination may occur transiently when hemoconcentration elevates hemoglobin disproportionately relative to albumin—for example, during aggressive fluid removal—while absolute albumin mass is simultaneously increasing due to synthesis or infusion effects. Its low frequency in this cohort (patients receiving no exogenous albumin) underscores its context-dependence.

This framework enables the identification of distinct albumin homeostasis phenotypes and their temporal evolution. Because the underlying mechanisms cannot be directly isolated without dedicated kinetic measurements, these classifications are hypothesis-generating rather than mechanistically definitive.

### 2.5. Statistical Analysis

The statistical analysis was designed to characterize the longitudinal relationship between the TER-like index and NAL and to test the hypothesis of temporal uncoupling between capillary permeability normalization and intravascular albumin recovery. A hierarchical analytical framework was applied, integrating descriptive, population-level longitudinal, individual trajectory, and state transition analyses. Continuous variables were summarized as means ± standard deviation or medians (interquartile range), as appropriate, and categorical variables were summarized as counts and percentages. Between-group comparisons were performed using Student's t-test, Mann–Whitney U test, chi-squared test, or Fisher’s exact test. Associations between the TER-like index and NAL were explored using Spearman’s rank correlation. Temporal trajectories were analyzed using generalized estimating equations (GEE) with robust standard errors to account for within-patient correlation and unequal numbers of repeated observations. Time was modeled as a categorical variable. TER-like index and NAL were first examined as separate continuous outcomes. To evaluate dynamic coupling, NAL was modeled as the dependent variable with the TER-like index as a time-dependent covariate, including a TER-by-time interaction term. Baseline SOFA score and 30-day mortality were assessed as potential effect modifiers. To explore inter-individual heterogeneity, patient-specific slopes of the TER-like index and NAL were estimated using linear regression across available time points. For instantaneous coupling analysis, observations were classified into four TER–NAL states based on the sign of each index. The probability of the uncoupled state (TER-like index ≤ 0 and NAL < 0) over time was analyzed using GEE with a logit link. Transitions between consecutive states were assessed using McNemar’s test. All analyses were performed using Stata 18.0 (StataCorp, College Station, TX, USA). Tests were two-sided, and statistical significance was set at *p* < 0.05. Given the hypothesis-generating nature of the study, no correction for multiple comparisons was applied; all *p*-values should therefore be interpreted in the context of exploratory, descriptive inference rather than confirmatory testing, and findings require independent prospective validation.

## 3. Results

A total of 389 patients were enrolled in the study. The baseline characteristics are presented in Table 1.

The mean age was 71.6 years (SD 12.6), and most patients were men (62.2%). Patients had a substantial comorbidity burden (mean CCI 5.4), with arterial hypertension (63.5%) and diabetes (27.0%) being the most frequent conditions. At enrolment, the mean SOFA score was 4.5 (SD 2.1), and the mean APACHE II score was 12.8 (SD 4.8).

**Figure 1:**
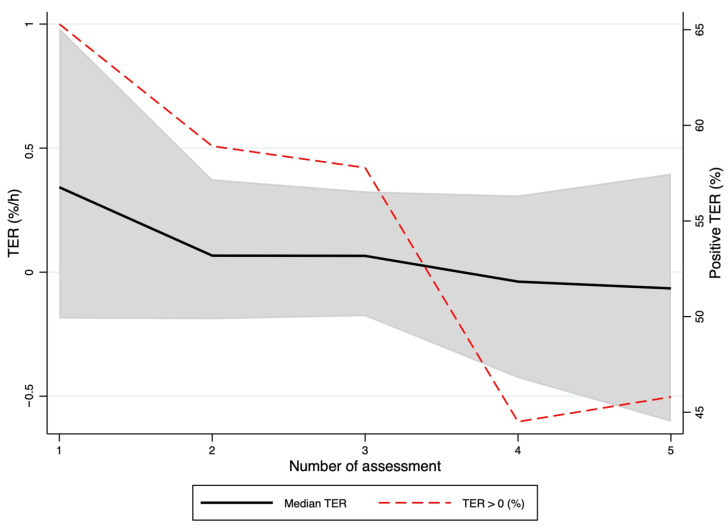
Time course of the TER-like index across five consecutive reassessments. The panel shows the distribution of TER-like index values at each reassessment, highlighting the median and interquartile dispersion. The figure also reports the proportion of observations with a positive TER-like index at each time point, underscoring the intra- and inter-individual variability of the phenomenon over successive clinical reassessments.

The time course of the TER-like index is shown in Figure 1. Median TER-like index was +0.34%/h (IQR, 1.17) at the first reassessment, +0.07%/h (IQR, 0.56) at the second, +0.07%/h (IQR, 0.50) at the third, −0.04%/h (IQR, 0.74) at the fourth, and −0.07%/h (IQR, 1.00) at the fifth reassessment. The proportion of patients with a positive TER-like index decreased from 65.3% at the first reassessment to 45.8% at the fifth reassessment (Supplementary Table S1). Overall, the temporal pattern of the TER-like index suggests an early phase characterized by a greater relative decline in albumin compared with hemoglobin, followed by progressive attenuation at subsequent reassessments, with values tending to stabilize in the later phases of follow-up (Figure 1).

**Figure 2:**
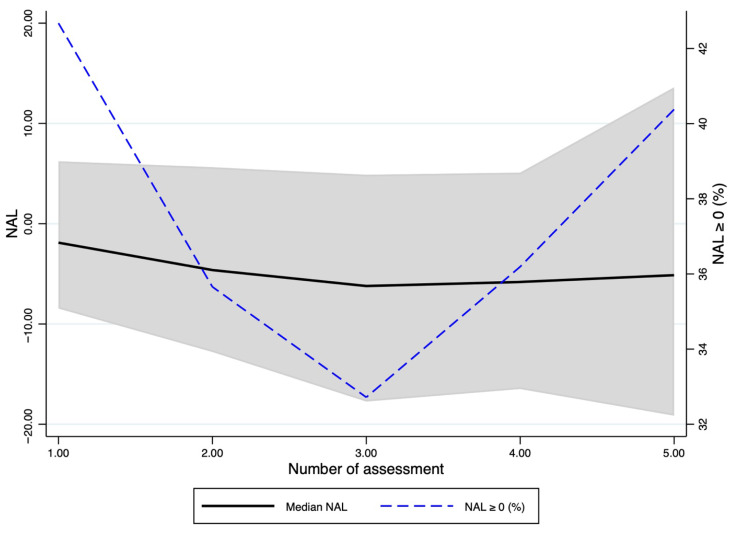
Time course of NAL across five consecutive reassessments. The figure depicts the distribution of NAL values at each time point with the median and interquartile range. The proportion of observations with a non-negative net albumin balance (NAL ≥ 0) at each reassessment was also reported, reflecting the marked heterogeneity of albumin balance profiles over time.

The time course of NAL is shown in Figure 2. At the first assessment, the median NAL was −1.88 g/L (IQR 14.69) and −4.62 g/L (IQR 18.43) at the second, −6.21 g/L (IQR 22.62) at the third, −5.80 g/L (IQR 21.58) at the fourth, and −5.13 g/L (IQR 32.75) at the fifth reassessment. The proportion of observations with NAL ≥ 0 was 42.7% at the first reassessment, 35.7% at the second, 32.7% at the third, 36.2% at the fourth, and 40.4% at the fifth, with an overall value of 37.5% across the entire observation period (Supplementary Table S1). Overall, the temporal pattern of NAL indicates the presence of net albumin loss in the early phases of follow-up, which persists across subsequent reassessments, with wide variability of values over time (Figure 2).

Across the available longitudinal observations (*n* = 1897), the TER-like index and NAL showed a significant inverse correlation (Spearman’s ρ = −0.54; *p* < 0.001), indicating that higher TER-like index values were associated with a negative albumin balance. In the baseline GEE model, adjusted for time point, the TER-like index was longitudinally associated with NAL (β = −7.46 g/L for each 1%/h increase in TER; 95% CI −8.69 to −6.22; *p* < 0.001). The time point also showed an independent effect, with a progressive reduction in NAL relative to the first assessment, reaching statistical significance from the second through the fourth reassessment. The inclusion of a TER-by-time interaction term demonstrated a time-heterogeneous relationship between NAL and the TER-like index (*p* for interaction <0.01). The effect of the TER-like index on NAL differed significantly across reassessments, indicating a temporal modification of the TER–NAL relationship. Among observations with TER-like index ≤ 0 (861/1897; 45.4%), NAL showed wide variability (median 3.6 g/L; IQR −6.3 to 17.0), and 40.7% of these observations were still associated with a net albumin loss (NAL < 0).

**Figure 3:**
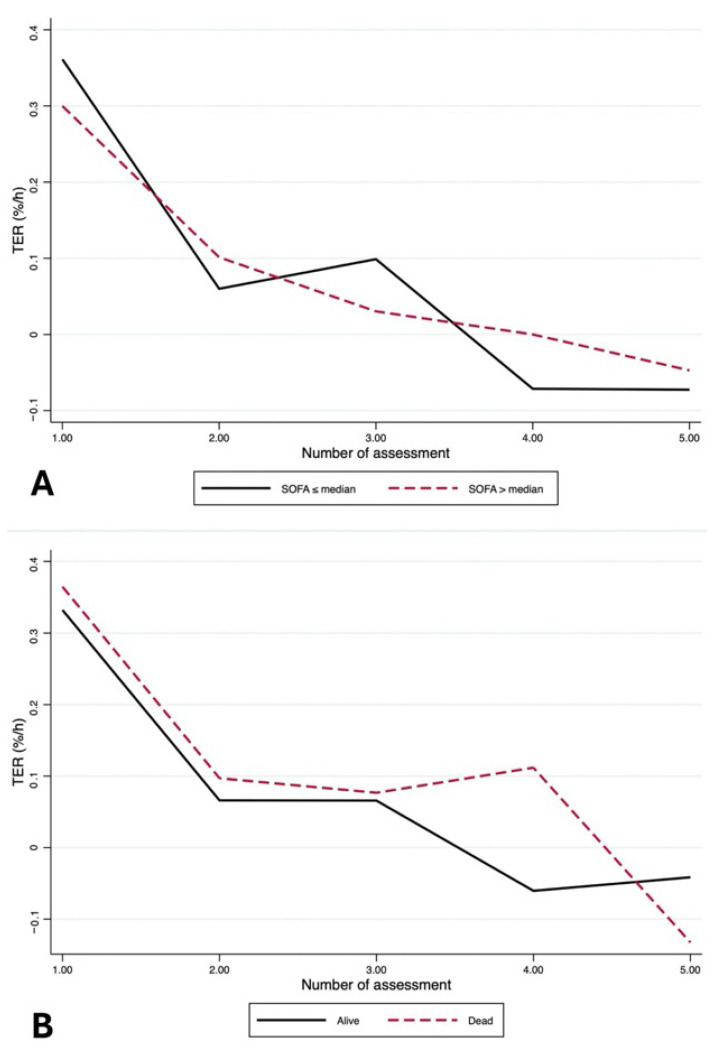
(**A**) Time course of the TER-like index across clinical reassessments, stratified by baseline severity (SOFA ≤ median vs. SOFA > median). (**B**) Time course of the TER-like index across clinical reassessments, stratified by outcome (survivors vs. non-survivors).

TER-like index showed a marked early decline over successive clinical reassessments, with largely overlapping temporal profiles in patients with SOFA ≤ median and SOFA > median, and a progressive convergence of values at later assessments (Figure 3A). Similarly, no differences in the TER-like index trajectories across reassessments were observed between patients who died and those who survived (Figure 3B).

In longitudinal GEE models, the TER-like index was not associated with baseline clinical severity or 30-day mortality after adjusting for time, including in a model that simultaneously included both variables. Across all models, time was the only significant determinant of TER, with a consistent progressive reduction across successive reassessments (Supplementary Table S2).

**Figure 4:**
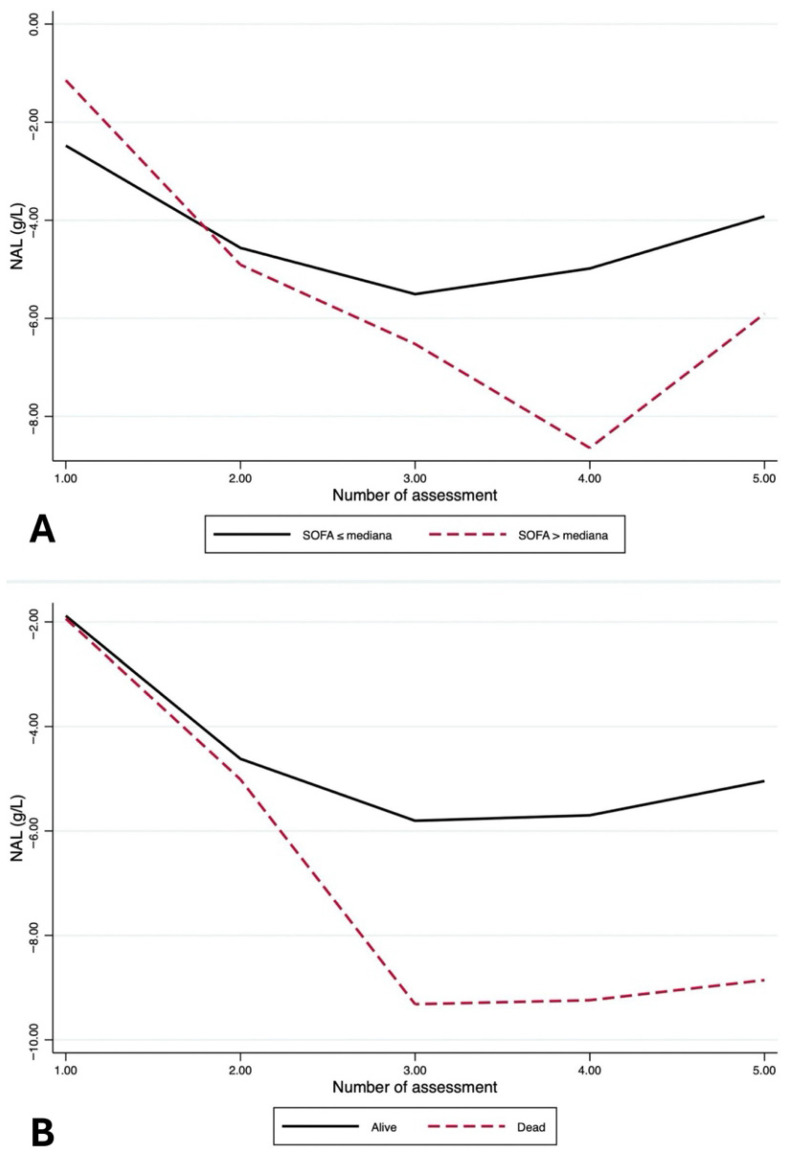
(**A**) Time course of NAL across clinical reassessments, stratified by baseline severity (SOFA ≤ median vs. SOFA > median). (**B**) Time course of NAL across clinical reassessments, stratified by outcome (survivors vs. non-survivors).

The time course of NAL according to baseline severity and 30-day outcomes is shown in Figure 4A,B.

In patients with SOFA > median, NAL was consistently more negative than in those with SOFA ≤ median across all reassessments, indicating a more pronounced and persistent negative albumin balance over time (Figure 4A). Likewise, when stratified by outcome, non-survivors showed more negative NAL values than survivors, with divergence evident from the earliest reassessments and persisting over time (Figure 4B). However, in time-adjusted longitudinal GEE models, NAL was not significantly associated with baseline severity or 30-day mortality (Supplementary Table S2). Regarding TER, time remained the only factor significantly associated with NAL, indicating a longitudinal change in albumin balance over reassessments independent of baseline severity and 30-day outcomes (Supplementary Table S2).

When considering the NAL and TER-like indices simultaneously, the association between the NAL and TER-like indices appeared independent of baseline severity and outcomes. Longitudinal GEE models adjusted for reassessments confirmed that the statistically significant TER–NAL relationship remained essentially unchanged after adjustment for baseline severity and 30-day mortality, which were not independently associated with longitudinal NAL values. In the fully adjusted model, the TER-like index was the only clinically relevant determinant of NAL, independent of time, baseline severity, and 30-day outcomes (Supplementary Table S2).

**Figure 5:**
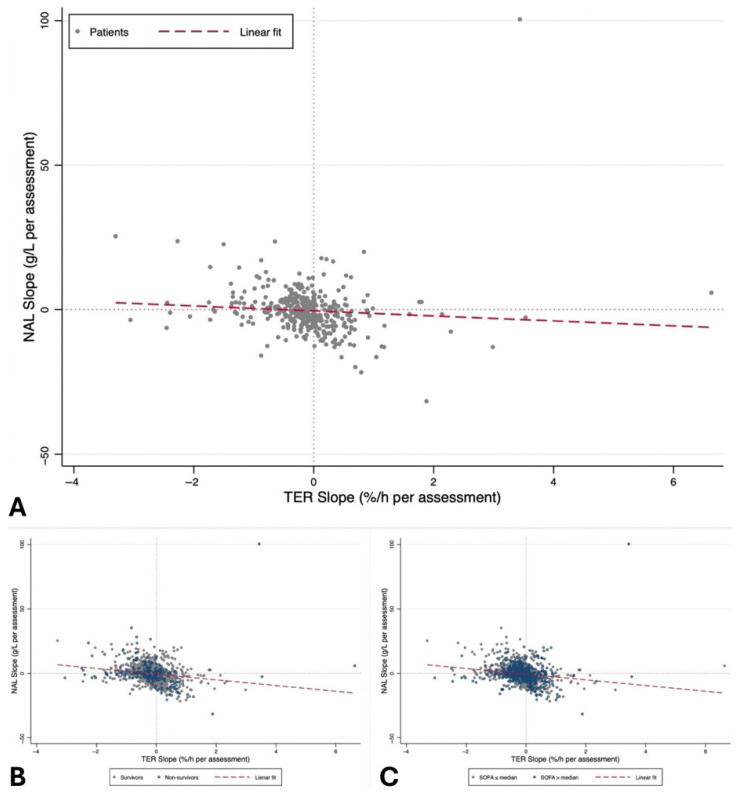
Patient-level uncoupling between the TER-like index and NAL. Scatter plot of patient-level temporal slopes of the TER-like index and NAL, estimated using linear regression on the number of clinical reassessments. (**A**) Overall relationship between the TER-like index slope and NAL slope. (**B**) Relationship between the TER-like index slope and NAL slope stratified by 30-day outcomes. (**C**) Relationship between the TER-like index slope and NAL slope stratified by baseline clinical severity (SOFA ≤ median vs. SOFA > median). The coefficient of determination for the overall linear fit (panel A) was R^2^ = 0.007 (*p* = 0.106), indicating a negligible linear association between patient-level TER-like index slopes and NAL slopes and quantitatively supporting the temporal uncoupling described in the text.

The dynamic TER–NAL relationship was further examined at the individual patient level using temporal slopes and joint distributions. The distribution of TER-like index slopes showed marked asymmetry, with a median of −0.13%/h per reassessment (IQR −0.31 to 0.08), indicating a progressive attenuation of the phenomenon in most patients, without significant differences by baseline severity or outcome (Supplementary Table S3) (Figure 5A).

In contrast, NAL slopes showed marked inter-individual heterogeneity and a clear dependence on baseline severity. Overall, the median NAL slope was −0.61 g/L per reassessment (IQR −3.50 to 3.66), with a significantly more negative longitudinal albumin balance in patients with higher baseline severity. This difference was not observed when stratifying by outcome (*p* = 0.95). Overall, patient-level slope analyses highlight a clear uncoupling between the TER-like index and NAL dynamics: the TER-like index exhibits a relatively uniform temporal decline that is independent of baseline severity and outcome, whereas NAL shows divergent trajectories strongly influenced by baseline patient severity (Figure 5B,C).

The assessment of the dynamic interconnection between the TER-like index and NAL, defined according to the sign of the TER-like index (>0 vs. ≤0) and NAL (≥0 vs. <0), across reassessments is reported in Supplementary Table S4.

Across reassessments, a temporal evolution in TER-like index–NAL patterns emerged. In particular, the proportion of observations in the TER-like index ≤0/NAL < 0 quadrant progressively increased, indicating an increasing frequency of conditions in which a negative albumin balance persisted despite reduction or normalization of the TER-like index (Supplementary Table S4). At the first reassessment, only 2.3% of patients were in the TER-like index ≤0/NAL < 0 quadrant, whereas at the last reassessment, this condition was observed in 22.6%. Overall, 22.4% of patients transitioned from another pattern toward the TER-like index ≤0/NAL < 0 quadrant, whereas only 2.1% showed the inverse transition (McNemar test, χ^2^ = 65.7; *p* < 0.001). The occurrence of a negative albumin balance in the presence of a TER-like index ≤0 therefore represents a frequent and non-random dynamic phenomenon during sepsis. Overall, the TER-like index ≤0/NAL < 0 quadrant accounted for 18.5% of all longitudinal observations and showed a clear temporal expansion. This pattern suggests that reduction or normalization of albumin escape does not necessarily translate into recovery of net balance, which may remain negative in a substantial proportion of observations and patients.

In the GEE model, the average longitudinal probability of observing the TER-like index ≤ 0/NAL < 0 quadrant was approximately 18% of observations (OR 0.23; 95% CI 0.18–0.29; *p* < 0.001), indicating that this condition represents a frequent and structural state during the clinical course, rather than an event restricted to specific temporal phases. In the longitudinal GEE model with a binary outcome (TER-like index ≤ 0/NAL < 0 vs. other quadrants), adjusted for within-patient correlation, membership in this quadrant was strongly associated with time point (Wald χ^2^ = 69.8; *p* < 0.001). Specifically, compared with the first reassessment, the odds of observing a normalized TER-like index with persistent net albumin loss increased markedly and progressively (R2: OR 9.2; R3: OR 11.5; R4: OR 16.8; R5: OR 12.6; all *p* < 0.001; Table 2). Stratified analyses by reassessment showed that, although this condition is not uniformly distributed over time, it increases sharply and progressively across successive reassessments. Overall, these findings indicate that normalization of albumin escape is not accompanied by recovery of net intravascular albumin balance in most patients, which remains frequently negative even when the TER-like index is ≤0.

## 4. Discussion

In this prospective longitudinal study of 389 intermediate-care patients with sepsis, albumin homeostasis dynamics were characterized using indices derived from routine laboratory parameters. Within a real-world clinical context and extending beyond purely experimental tracer-based models, to our knowledge, this is the largest cohort to systematically evaluate the longitudinal relationship between a ratio-based albumin kinetic index and net intravascular albumin balance. Intravascular albumin balance, conceived as the dynamic interplay between loss and restoration, determines the effective circulating protein pool and may serve as an integrated marker of permeability integrity and recovery mechanisms across the vascular, interstitial, and lymphatic compartments during sepsis [10,14,16]. Although the TER-like index and NAL were moderately correlated (ρ = −0.54, *p* < 0.001), their association was time-dependent (interaction *p* < 0.01). Joint longitudinal analysis revealed a progressive dissociation pattern and stabilization or improvement of the ratio (index ≤0) despite a persistent negative albumin balance (NAL < 0), observed in 22.6% of measurements by day 5. These findings indicate that attenuation of the TER-like index does not necessarily translate into recovery of intravascular albumin mass, supporting the concept of functional uncoupling between the relative albumin–hemoglobin ratio trajectory and restoration of effective protein homeostasis. This dissociation likely reflects heterogeneous contributions of synthesis, redistribution, and recovery mechanisms, underscoring the need for an integrated evaluation of ratio-based and mass-based indices in clinical sepsis.

Albumin homeostasis in sepsis reflects the integrated balance of processes operating across the vascular, interstitial, and lymphatic compartments [10,14,16]. Although increased transcapillary albumin extravasation due to endothelial glycocalyx disruption and altered permeability is well established [12,13,26], mechanisms governing protein recovery from the interstitium to the vascular space remain less clearly characterized, particularly in intermediate-severity sepsis populations. Guyton’s concept of interstitial washdown describes a lymphatic-mediated defense mechanism that limits interstitial protein accumulation and edema by promoting protein return to the circulation [14], a process that may be compromised in sepsis through lymphatic dysfunction and altered interstitial dynamics [10,27]. In parallel, sepsis suppresses hepatic albumin synthesis and redirects amino acids toward acute-phase protein production, contributing to a negative albumin balance independent of permeability changes. From this perspective, intravascular albumin balance should not be interpreted solely as a marker of capillary leak but rather as the net result of dynamically interacting escape and recovery mechanisms, providing a more comprehensive pathophysiological framework [19,24,28].

This study has several noteworthy aspects. First, the ALB/HB ratio–based TER-like index peaked early (median +0.34%/h on day 1) and declined toward negative values by day 5 (median −0.07%/h), with positive values decreasing from 65.3% to 45.8%. This pattern was consistent across severity and mortality strata, with the time point emerging as the dominant predictor rather than the SOFA score or survival. Overall, the TER-like index dynamics followed a relatively uniform course in IMCU sepsis, marked by an early decline and subsequent stabilization, largely independent of baseline risk. However, physiological interpretation requires caution. Early ratio decline is consistent with preferential transcapillary albumin loss but may also reflect impaired synthesis or altered recovery kinetics. Similarly, ratio stabilization (TER-like index close to 0) does not indicate normalization of permeability (classical TER, 5–6%/h) but rather that albumin is no longer decreasing relative to hemoglobin, a state potentially driven by reduced leak, resumed synthesis, enhanced lymphatic return, hemoconcentration, or a combination [18,26,29]. The uniformity of this transition across outcome groups suggests a common phase of IMCU sepsis without identifying the dominant mechanism at the individual level. The absence of independent associations between the TER-like index and either SOFA or 30-day mortality in time-adjusted GEE models reflects the dominant role of the temporal dimension in the longitudinal regression framework: once time is accounted for, the residual variance attributable to severity or outcome is substantially attenuated. This pattern is consistent with the known mathematical structure of GEE when a dominant temporal trend is present and does not negate the clinical relevance of the observed descriptive associations.

Second, in contrast to the relatively uniform trajectories of the TER-like index, NAL displayed marked inter-individual variability and severity-dependent patterns. Patients with higher baseline SOFA scores and non-survivors showed more negative NAL values persistently, indicating that absolute intravascular albumin depletion correlates with systemic severity and adverse outcomes [30,31]. This association remained independent of the TER-like index, suggesting that NAL and TER-like index dynamics capture distinct, although partially overlapping, dimensions of albumin homeostasis. Importantly, in patients with higher severity, negative NAL often persisted despite stabilization of the TER-like index, indicating ongoing absolute albumin depletion in the absence of further relative ALB/HB decline. This dissociation may reflect insufficient hepatic synthesis, impaired lymphatic–interstitial recovery, proportional losses of albumin and hemoglobin maintaining ratio stability, or persistent volume contraction [10,14,17]. These mechanisms are physiologically plausible in severe sepsis and may coexist; however, the present methodology cannot determine their relative contribution without direct assessment of synthesis and lymphatic function [10,14].

Finally, combined-state analysis showed that the proportion of observations characterized by a stabilized TER-like index with a persistent negative NAL (TER-like index ≤0, NAL < 0) increased from 2.3% at day 1 to 22.6% at day 5, with asymmetric transitions favoring entry into this state (*p* < 0.001). Patient-level slope analyses confirmed that ratio stabilization could coexist with persistently negative or worsening NAL trajectories, particularly in more severe patients, indicating inter-individual heterogeneity and dynamic uncoupling between the ratio-based kinetic index and absolute albumin balance. Although this pattern does not permit definitive mechanistic attribution, it supports testable hypotheses. Persistent negative NAL despite ratio stabilization may reflect impaired lymphatic recovery, reduced hepatic synthesis, volume effects, or proportional protein losses, alone or in combination [32]. Its increasing frequency, directional progression, and association with severity underscore its clinical relevance. Mechanistic studies incorporating direct assessments of synthesis, permeability, and volume kinetics are needed to clarify its determinants.

Overall, these findings suggest that in sepsis, regulation of protein balance does not rely solely on the ability to limit escape but also requires preserved mechanisms of recovery from the interstitium. From this perspective, the lymphatic system and interstitial washdown represent potential pathophysiological targets warranting direct investigation in future mechanistic studies. Persistence of a negative NAL despite ratio stabilization may reflect impaired protein recovery contributing to sustained interstitial edema and organ dysfunction; however, this interpretation remains hypothesis-generating and cannot be confirmed from the present observational data. These observations suggest a potentially distinct phase of albumin homeostasis beyond the initial leak phase, the determinants and therapeutic relevance of which require prospective mechanistic evaluation [10,33,34].

The clinical implications of our findings indicate that albumin homeostasis cannot be captured by static albumin concentrations alone. The combined use of the TER-like index and NAL reveals clinically meaningful patterns, including dissociation between ALB/HB stabilization and persistent albumin mass depletion in the late phases. This suggests that permeability, synthesis, lymphatic recovery, and volume distribution interact dynamically and may be differentially impaired across disease phases. Clinically, these phenotypes may guide therapeutic strategies, as dissociation states could identify candidates for albumin or metabolic support, whereas recovery patterns may not require targeted intervention. These hypotheses warrant prospective validation in large multicenter cohorts.

### Limitations

This study had several limitations. First, the single-center design may limit the external validity of the present study. Our IMCU cohort represents intermediate-severity sepsis managed without routine albumin or colloid administration, and therefore, the findings may not directly generalize to higher-severity ICU populations or settings with different therapeutic protocols. Nevertheless, systematic prospective data collection, many longitudinal observations, and adherence to guideline-based management strengthen the internal validity and support the robustness of the observed patterns.

Second, the kinetic parameters used are derived rather than directly measured. The TER-like index does not replicate tracer-derived transcapillary escape rate measurements and integrates permeability, synthesis, recovery, and volume effects. Therefore, it should be interpreted as a clinically applicable surrogate of albumin kinetics rather than a direct measure of capillary permeability. Similarly, NAL relies on estimated plasma volume rather than direct volume measurement. Blood volume was estimated using Nadler’s equations derived from baseline anthropometric data and was assumed constant across serial assessments; in patients with sepsis, however, blood volume is dynamically influenced by fluid administration, third-space losses, and vasopressor-mediated redistribution. This fixed-BV assumption may introduce systematic error in IVAM and NAL calculations, particularly in later reassessments when cumulative fluid balance is most variable. These indices are conceptually linked to the underlying physiological mechanisms but remain indirect approximations, reflecting the practical constraints of routine clinical assessment.

Third, albumin homeostasis is intrinsically dynamic and influenced by therapeutic interventions during hospitalization. Fluid resuscitation strategies, vasopressor use, nutritional support, and organ support therapies may modulate both transcapillary loss and recovery mechanisms. Because the treatments were not randomized, their effects cannot be fully disentangled from the underlying pathophysiology. Specifically, although 24 h cumulative fluid balance was recorded at each reassessment, granular data on cumulative fluid volumes by type, vasopressor doses, duration, and nutritional protein intake were not available for time-varying covariate modeling. These variables may independently modulate both the ALB/HB ratio and intravascular albumin mass through dilutional, oncotic, and synthetic mechanisms, and their omission represents a limitation that future prospective studies should address by incorporating structured treatment documentation. However, the exclusion of exogenous albumin and synthetic colloid administration reduced a major potential iatrogenic confounder and allowed the evaluation of endogenous albumin regulation in real-world care.

Fourth, the study was not designed to establish definitive prognostic relationships between the TER-like index, NAL, and clinical outcomes. The high temporal variability of these parameters and their modulation by therapy make simple predictive associations unlikely. Prognostic validation would require dedicated analyses with earlier and more granular endpoints beyond 30-day mortality.

Finally, as in all longitudinal studies in critically ill populations, serial reassessments were conditioned by survival. Missing measurements due to early death or discharge may introduce informative dropout, particularly in later phases, as patients surviving to later reassessments may systematically differ from those lost to follow-up. Although GEE with robust standard errors partially mitigates this by using all available observations under a missing-at-random framework, it does not fully correct for informative dropout. Sensitivity analyses using inverse probability weighting or joint longitudinal-survival modeling were not performed and represent an important methodological extension for future work. Findings from later reassessments (days 4–5) should therefore be interpreted with appropriate caution.

## 5. Conclusions

In septic patients, intravascular albumin balance emerges as the result of a dynamic equilibrium between loss and the capacity for restoration. Albumin escape appears to be a predominantly early and common phenomenon, whereas persistence of a negative net balance reflects vulnerability of re-equilibration mechanisms, more evident in clinically more compromised patients. Normalization of the TER-like index does not necessarily coincide with restoration of protein homeostasis, highlighting a temporal dissociation between the attenuation of relative albumin loss and the restoration of absolute intravascular albumin mass, the mechanisms of which remain to be directly elucidated. From this perspective, NAL represents a clinically accessible indicator of a complex pathophysiology integrating microvascular permeability, interstitial dynamics, and protein recovery. A dynamic, integrated assessment of the TER-like index and NAL may enable a more accurate pathophysiological reading of sepsis and, in the future, help guide therapeutic strategies targeting recovery and compartmental re-equilibration mechanisms beyond traditional macrohemodynamic interpretations. These observations are descriptive and hypothesis-generating in nature; prospective multicenter validation with pre-specified prognostic endpoints is required before clinical application of these indices can be recommended.

## Figures and Tables

**Table 1:** Demographic, medical history, and clinical characteristics of the study population.

Variable	Value
Patients, *n* (%)	389
Age, years, mean (SD)	71.6 (12.6)
Sex, *n* (%) Female Male	147 (37.8) 242 (62.2)
Weight, kg, mean (SD)	75.1 (16.6)
BMI, mean (SD)	26.2 (5.5)
Medical history, *n* (%) Hypertension Ischemic heart disease Chronic heart failure Stroke/TIA COPD Chronic renal failure Diabetes	247 (63.5) 52 (13.4) 58 (14.9) 33 (8.5) 49 (12.6) 73 (18.8) 105 (27)
Charlson Comorbidity Index, point, mean (SD)	5.4 (2.9)
Lactate, median (IQR)	1.7 (1.2–2.8)
SOFA score, point, mean (SD)	4.5 (2.1)
APACHE II, point, mean (SD)	12.8 (4.8)

**Table 2:** Longitudinal generalized estimating equation (GEE) analysis of the TER-like index ≤ 0/NAL < 0 quadrant. Results of GEE models with a binary outcome evaluating the probability of observing the TER-like index ≤ 0/NAL < 0 quadrant versus the other quadrants across clinical reassessments, accounting for within-patient correlation.

Time-Point Model (Reference: Reassessment 1)					
Variable	Beta(logit)	Robust SE	OR	95% CI	*p*-value
Reassessment 2 vs. 1	2.21	0.37	9.2	4.4–19	<0.001
Reassessment 3 vs. 1	2.44	0.36	11.5	5.7–23.2	<0.001
Reassessment 4 vs. 1	2.82	0.36	16.8	8.4–33.9	<0.001
Reassessment 5 vs. 1	2.54	0.36	12.6	6.2–25.7	<0.001
Intercept	−3.74	0.34	-	-	<0.001
Overall Model (without time points)					
Variable	Beta(logit)	Robust SE	OR	95% CI	*p*-value
Overall	−1.48	0.11	0.23	0.18–0.29	<0.001

## Data Availability

Data are only available on request due to privacy/ethical restrictions.

## Supplementary Materials

The following supporting information can be downloaded at: https://www.mdpi.com/article/10.3390/jcm15062427/s1, Table S1: Time course of TER-like index and NAL across reassessments; Table S2: Results of GEE models for longitudinal TER-like index and NAL; Table S3: Temporal slopes of TER-like index and NAL were estimated at the individual patient level using linear regression on the number of clinical reassessments; Table S4: Distribution of TER-like index–NAL quadrants across reassessments.

## Abbreviations

The following abbreviations are used in this manuscript:

ALB/HB	Albumin-to-hemoglobin ratio
APACHE II	Acute Physiology and Chronic Health Evaluation II
BMI	Body mass index
BV	Blood volume
CCI	Charlson Comorbidity Index
CI	Confidence interval
COPD	Chronic obstructive pulmonary disease
ED	Emergency department
GEE	Generalized estimating equations
HCT	Hematocrit
IMCU	Intermediate care unit
IQR	Interquartile range
IVAM	Intravascular albumin mass
NAL	Net albumin leakage
OR	Odds ratio
PV	Plasma volume
SOFA	Sequential Organ Failure Assessment
TER	Transcapillary escape rate

## References

[1] Arina P., Hofmaenner D.A., Singer M. (2024). Definition and Epidemiology of Sepsis. Semin. Respir. Crit. Care Med..

[2] Prescott H.C., Posa P.J., Dantes R. (2023). The Centers for Disease Control and Prevention’s Hospital Sepsis Program Core Elements. JAMA.

[3] Bauer M., Gerlach H., Vogelmann T., Preissing F., Stiefel J., Adam D. (2020). Mortality in sepsis and septic shock in Europe, North America and Australia between 2009 and 2019-results from a systematic review and meta-analysis. Crit. Care.

[4] La Via L., Sangiorgio G., Stefani S., Marino A., Nunnari G., Cocuzza S., La Mantia I., Cacopardo B., Stracquadanio S., Spampinato S. (2024). The Global Burden of Sepsis and Septic Shock. Epidemiologia.

[5] Woodcock T.E., Woodcock T.M. (2012). Revised Starling equation and the glycocalyx model of transvascular fluid exchange: An improved paradigm for prescribing intravenous fluid therapy. Br. J. Anaesth..

[6] Turcato G., Zaboli A., Filippi L., Cipriano A., Ferretto P., Maggi M., Lucente F., Marchetti M., Ghiadoni L., Wiedermann C.J. (2025). Endothelial Damage in Sepsis: The Interplay of Coagulopathy, Capillary Leak, and Vasoplegia-A Physiopathological Study. Clin. Pract..

[7] Turcato G., Zaboli A., Filippi L., Lucente F., Maggi M., Cipriano A., Marchetti M., Milazzo D., Wiedermann C.J., Ghiadoni L. (2025). Hemodynamic Heterogeneity in Community-Acquired Sepsis at Intermediate Care Admission: A Prospective Pilot Study Using Impedance Cardiography. Healthcare.

[8] Malbrain M.L., Marik P.E., Witters I., Cordemans C., Kirkpatrick A.W., Roberts D.J., Van Regenmortel N. (2014). Fluid overload, de-resuscitation, and outcomes in critically ill or injured patients: A systematic review with suggestions for clinical practice. Anaesthesiol Intensive Ther..

[9] Bharwani A., Dionne J.C., Pérez M.L., Englesakis M., Meyhoff T.S., Sivapalan P., Zampieri F.G., Wilcox M.E. (2025). Conservative versus liberal fluid resuscitation for septic patients at risk for fluid overload: A systematic review with meta-analysis. J. Crit. Care.

[10] Dull R.O., Hahn R.G. (2025). Physiology and Molecular Mechanisms of the “Third Fluid Space”. J. Clin. Med..

[11] Gradel K.O., Vinholt P.J., Magnussen B., Pedersen C., Jensen T.G., Kolmos H.J., Lassen A.T. (2018). Hypoalbuminaemia as a marker of trans-capillary leakage in community-acquired bacteraemia patients. Epidemiol. Infect..

[12] Juffermans N.P., Radermacher P., Joffre J. (2026). Vascular permeability and loss of glycocalix in sepsis: The role of fluid resuscitation. Intensive Care Med..

[13] McMullan R.R., McAuley D.F., O’Kane C.M., Silversides J.A. (2024). Vascular leak in sepsis: Physiological basis and potential therapeutic advances. Crit. Care.

[14] Hahn R.G., Dull R.O. (2021). Interstitial washdown and vascular albumin refill during fluid infusion: Novel kinetic analysis from three clinical trials. Intensive Care Med. Exp..

[15] Ren D., Liu Y., Qin Y. (2025). Effects of Human Serum Albumin Infusion on Microcirculation, Capillary Leakage, and Endothelial Function in Sepsis Patients: A Retrospective Cohort Study. Shock.

[16] Hansrivijit P., Yarlagadda K., Cheungpasitporn W., Thongprayoon C., Ghahramani N. (2021). Hypoalbuminemia is associated with increased risk of acute kidney injury in hospitalized patients: A meta-analysis. J. Crit. Care.

[17] Wiedermann C.J., Zaboli A., Lucente F., Filippi L., Maggi M., Ferretto P., Cipriano A., Voza A., Ghiadoni L., Turcato G. (2025). Temporal Decline in Intravascular Albumin Mass and Its Association with Fluid Balance and Mortality in Sepsis: A Prospective Observational Study. J. Clin. Med..

[18] Seldén D., Tardif N., Wernerman J., Rooyackers O., Norberg Å. (2025). Net albumin leakage in patients in the ICU with suspected sepsis. A prospective analysis using mass balance calculations. Crit. Care.

[19] Gattarello S., Gazzé G., Rollo E., Donati B., Caronna M., Grava I., Chiumiento C., Li Z., Gallese W., Nocera D. (2026). Albumin kinetics, intravascular fluid volume, and respiratory function in pigs ventilated at different levels of mechanical power following crystalloid vs. albumin infusion. Intensive Care Med. Exp..

[20] Levitt D.G., Levitt M.D. (2016). Human serum albumin homeostasis: A new look at the roles of synthesis, catabolism, renal and gastrointestinal excretion, and the clinical value of serum albumin measurements. Int. J. Gen. Med..

[21] Komáromi A., Estenberg U., Hammarqvist F., Rooyackers O., Wernerman J., Norberg Å. (2016). Simultaneous assessment of the synthesis rate and transcapillary escape rate of albumin in inflammation and surgery. Crit. Care.

[22] Chahid Y., Rorije N.M.G., El Boujoufi S., Mathôt R.A.A., Vogt L., Verberne H.J. (2021). Transcapillary escape rate of ^125^I-albumin in relation to timing of blood sampling: The need for standardization. EJNMMI Radiopharm. Chem..

[23] Singer M., Deutschman C.S., Seymour C.W., Shankar-Hari M., Annane D., Bauer M., Bellomo R., Bernard G.R., Chiche J.D., Coopersmith C.M. (2016). The Third International Consensus Definitions for Sepsis and Septic Shock (Sepsis-3). JAMA.

[24] Callum J., Skubas N.J., Bathla A., Keshavarz H., Clark E.G., Rochwerg B., Fergusson D., Arbous S., Bauer S.R., China L. (2024). Use of Intravenous Albumin: A Guideline From the International Collaboration for Transfusion Medicine Guidelines. Chest.

[25] Erstad B.L. (2020). The Revised Starling Equation: The Debate of Albumin Versus Crystalloids Continues. Ann. Pharmacother..

[26] Reed R.K., Rubin K. (2010). Transcapillary exchange: Role and importance of the interstitial fluid pressure and the extracellular matrix. Cardiovasc. Res..

[27] Stewart R.H. (2020). A Modern View of the Interstitial Space in Health and Disease. Front. Vet. Sci..

[28] Rothschild M.A., Oratz M., Schreiber S.S. (1973). Albumin metabolism. Gastroenterology.

[29] Moshage H.J., Janssen J.A., Franssen J.H., Hafkenscheid J.C., Yap S.H. (1987). Study of the molecular mechanism of decreased liver synthesis of albumin in inflammation. J. Clin. Investig..

[30] Touma E., Bisharat N. (2019). Trends in admission serum albumin and mortality in patients with hospital readmission. Int. J. Clin. Pract..

[31] Jellinge M.E., Henriksen D.P., Hallas P., Brabrand M. (2014). Hypoalbuminemia is a strong predictor of 30-day all-cause mortality in acutely admitted medical patients: A prospective, observational, cohort study. PLoS ONE.

[32] Vincent J.L., Dubois M.J., Navickis R.J., Wilkes M.M. (2003). Hypoalbuminemia in acute illness: Is there a rationale for intervention? A meta-analysis of cohort studies and controlled trials. Ann. Surg..

[33] Tang F., Zhao X.L., Xu L.Y., Zhang J.N., Ao H., Peng C. (2024). Endothelial dysfunction: Pathophysiology and therapeutic targets for sepsis-induced multiple organ dysfunction syndrome. Biomed. Pharmacother..

[34] Pruitt L.G. (2020). Lymphatic flow modulation as adjunct therapy for septic shock. Med. Hypotheses.

