# Supplementary material for: Temporal Dissociation Between Intravascular Albumin Mass and Transcapillary Escape Dynamics in Sepsis: A Longitudinal Characterization of Albumin Homeostasis Using Routine Laboratory Parameters
Source: J Clin Med. 2026 Mar 22;15(6):2427. doi: 10.3390/jcm15062427 (PMC13026996; doi:10.3390/jcm15062427)
Supplement: Supplementary file 1 [file jcm-15-02427-s001.zip › jcm-4191744-supplementary.pdf]

Supplementary Table S1: Time course of TER-like index and NAL across reassessments

Reassessment	Number	TER-like index, %/h, median (IQR)	TER-like index >0 (%)	NAL, g/L, median (IQR)	NAL>0 (%)
1	389	0.34 (1.17)	65.3	-1.88 (14.69)	42.7
2	387	0.07 (0.56)	58.9	-4.62 (18.43)	35.7
3	379	0.07 (0.50)	57.8	-6.21 (22.62)	32.7
4	373	-0.04 (0.74)	44.5	-5.80 (21.58)	36.2
5	369	-0.07 (1.00)	45.8	-5.13 (32.75)	40.4
Totale	1897	0.06 (0.76)	-	-1.01 (15.31)	37.5

Supplementary Table S2. Results of GEE models for longitudinal TER-like index and NAL. Regression coefficients (β) with 95% confidence intervals (95% CI) and p values are reported for predictors included in each model, accounting for within-patient correlation and modeling time as a categorical variable.

Model	Outcome	Predictors	β (IC 95%)	p-value
GEE-TER-1	TER-like index	SOFA / Time	SOFA: 0.04 (-0.04 ; 0.13)	0.337
GEE-TER-2	TER-like index	Outcome / Time	Outcome: 0.05 (-0.08 ; 0.1)	0.405
GEE-TER-3	TER-like index	SOFA / Outcome / Time	SOFA: 0.04 (-0.04 ; 0.12)<br>Outcome: 0.05 (-0.08 ; 0.18)	0.366<br>0.435
GEE-NAL-1	NAL	SOFA / Time	SOFA: -0.22 (-3.86 ; 3.4)	0.903
GEE-NAL-2	NAL	Outcome / Time	Outcome: -0.72 (-5.81; 4.3)	0.781
GEE-NAL-3	NAL	SOFA / Outcome / Time	SOFA: -0.17 (-3.82; 3.4)<br>Outcome: -0.69 (-5.81; 4.4)	0.929<br>0.789
GEE-NAL-TER-1	NAL	TER-like index / SOFA / Time	TER: -7.45 (-8.69 ; -6.2)<br>SOFA: 0.03 (-3.11 ; 3.1)	0.008<br>0.983
GEE-NAL-TER-2	NAL	TER-like index / SOFA / Outcome / Time	TER: -7.46 (-8.69 ; -6.2)<br>Outcome: -0.92 (-5.03 ; 3.1)	<0.001
GEE-NAL-TER-2	NAL	TER-like index / SOFA / Outcome / Time	TER: -7.46 (-8.69 ; -6.2)<br>SOFA: 0.11 (-3.05 ; 3.2)<br>Outcome: -0.94 (-5.08 ; 3.1)	<0.001<br>0.943<br>0.655

Supplementary Table S3: Temporal slopes of TER-like index and NAL were estimated at the individual patient level using linear regression on the number of clinical reassessments. Slopes are reported as median (IQR) overall and stratified by baseline clinical severity (baseline SOFA) and 30-day outcome, with corresponding between-group comparisons.

Endpoint (patient-level)	Groups	Median slope (group 1)	Median slope (group 2)	p-value
TER-like index slope	Overall	−0.13 (−0.31 ; 0.08)		-
TER-like index slope	SOFA	−0.13 (−0.29 ; 0.05)	−0.13 (−0.32 ; 0.14)	0.47
TER-like index slope	Outcome	−0.13 (−0.30 ; 0.07)	−0.20 (−0.42 ; 0.11)	0.10
TER-like index slope	Overall	−0.61 (−3.50 ; 3.66)		-

NAL slope	SOFA	−0.36 (−3.06 ; 3.84)	−0.85 (−3.75 ; 2.66)	0.03
NAL slope	Outcome	−0.57 (−3.45 ; 3.05)	−0.63 (−3.52 ; 4.16)	0.95

Supplementary Table S4. Distribution of TER-like index–NAL quadrants across reassessments. Distribution of observations across the four quadrants defined by the combination of TER-like index and NAL over the five clinical reassessments. Data are reported as number of observations and row percentage.

Reassessment	TER-like index >0 / NAL≥0	TER-like index>0 / NAL<0	TER-like index≤0 / NAL≥0	TER-like index≤0 / NAL<0	Total
1	214 (55)	9 (2.3)	40 (10.3)	126 (32.4)	389
2	180 (46.5)	69 (17.8)	48 (12.4)	90 (23.3)	387
3	174 (45.9)	81 (21.4)	45 (11.9)	79 (20.8)	379
4	132 (35.4)	106 (28.4)	34 (9.1)	101 (27.1)	373
5	135 (36.6)	85 (23)	34 (9.2)	115 (31.2)	369
Total	835 (44)	350 (18.5)	201 (10.6)	511 (26.9)	1897
